# Endocrine biomarkers related to sleep-wake cycle and sleep disturbances in patients with bipolar disorder: A systematic review

**DOI:** 10.1192/j.eurpsy.2022.914

**Published:** 2022-09-01

**Authors:** L. Gonzalez-Blanco, C. Moya Lacasa, S. Jiménez-Fernández, M. Valtueña, C. Martinez-Cao, A. García Fernández, P.A. Saiz, M.P. Garcia-Portilla, J. Bobes, L. Gutierrez Rojas

**Affiliations:** 1 University of Oviedo, Department Of Psychiatry, oviedo, Spain; 2 Servicio de Salud del Principado de Asturias, Psychiatry, Oviedo, Spain; 3 Instituto de Investigación Sanitaria del Principado de Asturias, Psychiatry, Oviedo, Spain; 4 Instituto de Neurociencias del Principado de Asturias, Psychiatry, Oviedo, Spain; 5 Centro de Investigación Biomédica en Red de Salud Mental (CIBERSAM), Psychiatry, Oviedo, Spain; 6 Servicio de Salud del Principado de Asturias, Psiquiatría, Oviedo, Spain; 7 University of Oviedo, Department Of Psychiatry, Oviedo, Spain; 8 Complejo Hospitalario de Jaén, Unidad De Salud Mental Infanto Juvenil, Jaén, Spain; 9 Hospital Clínico San Cecilio, Psychiatry, Granada, Spain

**Keywords:** circadian rhythms, sleep disorders, orexin, melatonin

## Abstract

**Introduction:**

Sleep and circadian disturbances have been widely studied in patients with bipolar disorder (BD) (Duarte Faria et al., 2015; Gonzalez, 2014). However, there is no clear evidence about the role of peripheral biomarkers of circadian cycle in this population.

**Objectives:**

This systematic review aims to identify potential endocrine biomarkers of circadian rhythm in blood and study their relationship with sleep problems in BD.

**Methods:**

An electronic search of Pubmed and PsycoInfo databases were performed. It includes articles about the topic from 1991 to 2021. The search strategy was: (“Peripheral biomarkers” OR “biological markers” OR biomarker OR cortisol OR melatonin OR orexin OR hypocretin) AND (blood OR serum OR plasma) AND (“sleep-wake” OR “circadian rhythm” OR sleep OR insomnia) AND “bipolar”.

**Results:**

92 records were obtained after excluding duplicates. Only five studies met the inclusion criteria (n = 499; BD = 125; unipolar depression = 148; schizophrenia = 80; controls = 146). The endocrine parameters analyzed were: cortisol (3 studies), melatonin (1 study) and orexin-A (1 study). Overall, no significant associations between these biomarkers and sleep disturbances, assessed with subjective (psychometric evaluation) and/or objective (polysomnography) measures, were detected.

**Conclusions:**

This systematic review highlights the lack of studies that explores the role of endocrine biomarkers related to circadian function in the pathophysiology of sleep disturbances in BD.

**Disclosure:**

No significant relationships.

